# Observations on the Treatment of Certain Severe Forms of Hæmorrhoidal Excrescence

**Published:** 1818-04

**Authors:** 


*Observations on the Treatment of certain severe Forms of Hæmorrhoidal Excrescence.* By JOHN KIRBY, A. B. London, 1817. pp. 56.

THE removal of hæmorrhoidal excrescences by the knife is by no means so rare an operation in this kingdom as Mr. Kirby imagines; but it may be less frequently performed than it should be. The disease is one, which, from personal knowledge, we can say, renders existence miserable; and consequently every attempt towards the relief of it must be proportionably valuable. The few cases from which is constructed the present pamphlet, are as follow,

*Case* 1. Mr. Kirby once witnessed the application of a ligature to a severe hæmorrhoidal excrescence in a female about 50 years of age, who was emaciated, and exhausted by pain and hæmorrhage. The anus readily admitted the two first fingers. The integuments of its border hung like a circular tube between the nates, to the extent of two inches, assuming the appearance of a prolapsus ani, excepting in its colour and the disposition of its rugæ. Whenever she made an effort, as at stool, a number of red, firm, circular tumours descended, and produced those painful consequences which it is unnecessary to describe. These tumours were severally included in ligatures. Peritoneal inflammation; convulsions; suppression of urine came on, and nearly proved fatal. In another case, the application of a ligature was followed by tetanus and death.

*Case* 2. Mr. P. ætat. 45, had a projection of integuments at the margin of the anus, of an inch and a half, terminating in a number of soft, livid, pile-like tumours. The prolapsus was great after each stool. His face was indicative of his sufferings. Mr. K. passed a large curved needle, carrying a strong ligature, through the entire circle of the pendulous projection. Directing the patient to force gently downwards, while Mr. K. drew the parts firmly towards him, they were removed by a single stroke of the scalpel. Several veins, which lay on the surface of the tumour, were divided, but quickly ceased to bleed. Two small arteries spouted, but were soon suppressed by the ends of the fingers, and a sponge supported by the T bandage. On the third day he dressed himself, and went to his usual occupations.

*Case* 3. Hayden, 40 years of age, tall, thin, and ghastly. Anus surrounded by a flaccid circular projection covered by the common integuments externally; lined internally by a vascular tunic. The finger introduced into the anus found the intestine every where occupied by soft round, elastic tumours, nearly painless. At stool a protrusion always took place, accompanied by acute pain and hæmorrhage. Hæmorrhage followed the operation from a small artery, which, however, was secured. He perfectly recovered.

*Case* 4. B——, Esq. ætat. 40, a free liver, of sedentary habits, and subject to internal piles for many years. Has now a *fistula in ano*, with discharge. The integuments at the margin of the anus appear elongated to the extent of an inch and better; they are tender and excoriated. The fistula was first operated on, and then the pendulous border of the anus was removed, as in the other cases. Nothing troublesome ensued.

*Case* 5. A young gentleman, 21 years of age, had been subject to piles for twelve months, accompanied by considerable distress. Every time he went to stool a bunch of tumours descended, and required moderate pressure to effect their return, with the horizontal position. The integuments, at the margin of the anus, projected a little and were soft and flabby. The pendulous portions were removed, and the gentleman quite recovered.

The remainder of Mr. K's work is formed of quotations from, and observations on, the writings of Pott, Hey, Abernethy, &c. with several long extracts in French, from a work on Hæmorrhoids by Larrogue. Nothing is said on the internal treatment of piles themselves, nor on the connexion of their phænomena with certain states of the constitution; though we think our author might have more usefully filled the remainder of the work with these subjects, than with the disquisitions above mentioned.) Mr. K's work however will, doubtless, lead the attention of surgeons more to this painful and troublesome complaint.

---

